# Heterologous Expression of Two *Brassica campestris* CCCH Zinc-Finger Proteins in *Arabidopsis* Induces Cytoplasmic Foci and Causes Pollen Abortion

**DOI:** 10.3390/ijms242316862

**Published:** 2023-11-28

**Authors:** Liai Xu, Xingpeng Xiong, Tingting Liu, Jiashu Cao, Youjian Yu

**Affiliations:** 1 Key Laboratory of Quality and Safety Control for Subtropical Fruit and Vegetable, Ministry of Agriculture and Rural Affairs, Collaborative Innovation Center for Efficient and Green Production of Agriculture in Mountainous Areas of Zhejiang Province, College of Horticulture Science, Zhejiang A&F University, Hangzhou 311300, China; 20210035@zafu.edu.cn; 2 Laboratory of Cell & Molecular Biology, Institute of Vegetable Science, Zhejiang University, Hangzhou 310058, China; xiongxingpeng@jcut.edu.cn (X.X.); 11416009@zju.edu.cn (T.L.)

**Keywords:** CCCH zinc-finger proteins, cytoplasmic foci, pollen development, *Brassica campestris*, *Arabidopsis thaliana*, mRNA metabolism

## Abstract

The membrane-less organelles in cytoplasm that are presented as cytoplasmic foci were successively identified. Although multiple CCCH zinc-finger proteins have been found to be localized in cytoplasmic foci, the relationship between their specific localization and functions still needs further clarification. Here, we report that the heterologous expression of two *Brassica campestris* CCCH zinc-finger protein genes (*BcMF30a* and *BcMF30c*) in *Arabidopsis thaliana* can affect microgametogenesis by involving the formation of cytoplasmic foci. By monitoring the distribution of proteins and observing pollen phenotypes, we found that, when these two proteins were moderately expressed in pollen, they were mainly dispersed in the cytoplasm, and the pollen developed normally. However, high expression induced the assembly of cytoplasmic foci, leading to pollen abortion. These findings suggested that the continuous formation of BcMF30a/BcMF30c-associated cytoplasmic foci due to high expression was the inducement of male sterility. A co-localization analysis further showed that these two proteins can be recruited into two well-studied cytoplasmic foci, processing bodies (PBs), and stress granules (SGs), which were confirmed to function in mRNA metabolism. Together, our data suggested that BcMF30a and BcMF30c play component roles in the assembly of pollen cytoplasmic foci. Combined with our previous study on the homologous gene of *BcMF30a/c* in *Arabidopsis*, we concluded that the function of these homologous genes is conserved and that cytoplasmic foci containing *BcMF30a/c* may participate in the regulation of gene expression in pollen by regulating mRNA metabolism.

## 1. Introduction

Eukaryotes have evolved to have a universal mechanism, namely compartmentalization, to achieve spatial-temporal control over biochemical reactions in living cells. With the exception of membrane-bound organelles, an alternative strategy to attain compartmentalization involves the formation of membrane-less organelles, which are not delimited by membranes, but their composition, structure, and function are still well maintained. In recent years, membrane-less organelles were successively described both in nucleus and cytoplasm [1,2,3]. The membrane-less organelles in the cytoplasm appear as cytoplasmic foci in eukaryotic cells, and they are usually rich in messenger ribonucleoproteins (mRNPs) assembled from mRNAs and proteins; thereby, they are also called mRNP granules [4]. These granules have been demonstrated to regulate mRNA metabolism, including transcription, storage, decapping, and degradation, as well as translation, transport, and signal transduction [5,6,7,8]. These findings suggest that these mRNP granules may be widely involved in the regulation of gene expression and, thereby, participate in various growth and development processes of living organisms.

The two most studied mRNP granules in eukaryotes are cytoplasmic processing bodies (PBs) and stress granules (SGs), which are considered to be distinct yet closely related [9]. SGs are usually detected during a variety of stressful conditions [10]. PBs are a bit more constitutive than can be microscopically observed in unstressed cells, whereas stress stimuli can promote their assembly and make them larger [11]. Moreover, PBs and SGs are able to interact with each other [12,13]. As the protein compositions of various PBs and SGs are continuously identified, it is found that the functional classifications of their constituent proteins are very similar, which partially explains their functional relevance: both PBs and SGs are generally considered to be able to concentrate mRNAs dispersed in the cytoplasm and regulate their fate, either by storage, decay, or re-entering translation [9]. However, with the determination of the proteomes of PBs and SGs formed in various cell types or under different stresses, the compositional heterogeneities between PB-SG, PB-PB, and SG-SG were also revealed [14,15,16,17]. The characterization of the protein components of mRNP granules specific to cell types or stressors may help reveal mechanisms of how to fine-tune these granules to regulate gene expression to coordinate cell growth and development in specific situations.

In recent years, research on mRNP granules has been in full swing; although most studies still focus on animals and microorganisms, there have gradually been gratifying discoveries in plants [3]. It has been reported that UBP1b, HSP101, and VOZ2 are the components of heat-responsive SGs and participate in plant heat tolerance [18,19,20], while UBP1c is implicated in the reversible formation of hypoxia-responsive SGs [21]. DCP5, EIN2, and TZF9 are the components of PBs induced by dark, ethylene, and pathogen-associated molecular patterns, respectively [22,23,24,25]. In addition, it was found that a number of proteins can be localized in cytoplasmic foci similar to PBs and SGs, including several tandem zinc-finger proteins [26]. Zinc-fingers are classified into different types based on the conserved cysteine (C) and histidine (H) residues bound to zinc ions [27]. In Arabidopsis, the family of “CCCH-type” zinc-finger proteins contains 68 members, which can be roughly divided into tandem CCCH zinc-finger proteins (TZFs; containing two CCCH zinc-finger motifs) and non-TZFs [28]. Among the 26 TZFs, proteins containing a plant-unique TZF motif preceded by an arginine-rich (RR) domain were further defined as RR-TZF, known as AtTZFs (AtTZF1-AtTZF11) [29]. Remarkably, all the AtTZFs and another two TZFs (AtC3H14 and AtC3H15) were shown to be localized in cytoplasmic foci when they were heterologously and transiently expressed in maize protoplasts [26]. Further investigations showed that AtTZF1/4/5/6/9 can co-localize with marker proteins of PBs and SGs [29,30,31], suggesting that they may be components of these mRNP granules. However, with the exception of newly identified CCCH zinc-finger proteins AtC3H18 [32] and AtC3H18-Like (AtC3H18L) [33], few non-TZFs have been identified as cytoplasmic foci localization proteins. In addition, seven CCCH zinc-finger proteins were identified to be localized in the nucleus and presented as nucleoplasmic foci, and most of them were found to be involved in the regulation of mRNA metabolism as RNA-binding proteins [34,35,36,37,38,39,40].

Recently, we identified several CCCH zinc-finger protein genes that may be involved in pollen development in *Brassica campestris* [41]. A genetic analysis revealed that there are two paralogous genes, *BcMF30a* and *BcMF30c*, encoding non-TZFs, which are specifically expressed in pollen during microgametogenesis. Moreover, both the double knockout and overexpression of these two genes can cause pollen abortion due to degradation of pollen contents [41,42]. Interestingly, when *BcMF30a* and *BcMF30c* are transiently expressed in tobacco leaf epidermal cells, heat shock can induce the formation of cytoplasmic foci, which is similar to their *Arabidopsis* homologous gene [32,42]. However, can these two proteins be recruited into cytoplasmic foci in pollen? If so, it is still unclear whether the cytoplasmic foci positioning is related to their biological functions during pollen development. Actually, although it has been determined that all AtTZFs and several other CCCH zinc-finger proteins show the characteristics of localizing to cytoplasmic foci, there are still few studies demonstrating that this specific localization pattern is directly related to their functions. Here, respectively, we fused *BcMF30a* and *BcMF30c* with *GFP* translationally, and expressed them in Arabidopsis, driven by their native promoters. In this way, the phenotype of heterologous expression of *BcMF30a* and *BcMF30c* in Arabidopsis can be analyzed, and the performance of fusion proteins in intact plants can also be monitored. According to our data, it was found that a high expression of BcMF30a or BcMF30c in pollen can promote the assembly of cytoplasmic foci, and the continuous formation of these foci was the main inducer of pollen abortion. A co-localization analysis demonstrated that both BcMF30a and BcMF30c can co-localize with PB and SG marker proteins, which indicated that cytoplasmic foci containing these two non-TZFs may have similar functions to PBs and SGs and possibly participate in pollen development by regulating mRNA metabolism.

## 2. Results

### 2.1. Heterologous Expression of BcMF30a or BcMF30c in Arabidopsis Causes Different Pollen Phenotypes in the Same Anther

In order to be able to visually observe the expression of BcMF30a and BcMF30c in cells, we created translational fusion genes (*BcMF30a*/*BcMF30c* coding sequences-*GFP*) driven by their native promoters. Then, the *ProBcMF30a:BcMF30a-GFP* and *ProBcMF30c:BcMF30c-GFP* constructs were transferred into Arabidopsis so that we could obtain enough transgenic plants and their progenies for observation and analysis in a short period of time.

**Figure 1:**
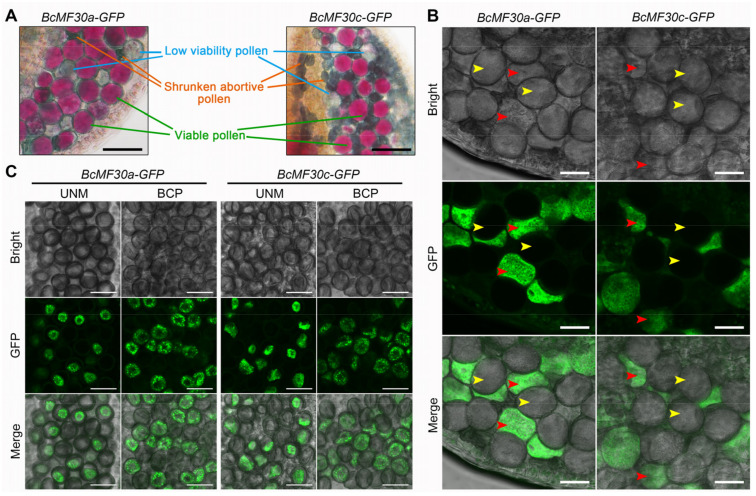
Cytological observation of pollen grains from T_1_ lines of *BcMF30a-GFP* and *BcMF30c-GFP* transgenic plants of *Arabidopsis thaliana*. (**A**) Alexander staining of mature pollen grains from representative T_1_ transgenic plants. The distribution of tricellular pollen grains (**B**), microspores, and bicellular pollen grains (**C**) expressing GFP fusion proteins in anthers from T_1_ lines of *BcMF30a-GFP* and *BcMF30c-GFP* plants. UNM, uninucleate microspore; BCP, bicellular pollen. Red arrows indicate transgenic pollen, and yellow arrows indicate wild-type pollen in (**B**). Bars = 50 μm in (**A**) and 20 μm in (**B**,**C**).

A total of 13 and 28 positive transgenic Arabidopsis plants expressing *BcMF30a* or *BcMF30c* heterologously were obtained, named *BcMF30a-GFP* (OE#1-13) and *BcMF30c-GFP* (OE#1-28), respectively. Interestingly, the Alexander staining results showed that all the T_1_ transgenic plants harbored varying numbers of normal pollen grains with high viability, normal-size pollen grains with low vigor, and aborted pollen with severe collapse in the same anther (Figure 1A). Moreover, in both *BcMF30a-GFP* and *BcMF30c-GFP* transgenic plants, GFP fluorescence signals were observed only in some but not in all mature pollens (Figure 1B). These findings indicated that both transgenic and wild-type pollen existed in the same anther and that wild-type pollen became a perfect control for transgenic pollen (Figure 1B). The co-existence of transgenic and wild-type pollen in the same anther was also observed at the uninucleate stage and the binucleate stage (Figure 1C). Remarkably, unlike wild-type pollen exhibiting a spindle morphology, those tricellular pollen grains expressing BcMF30a-GFP or BcMF30c-GFP proteins showed irregular morphology and even shrinkage (Figure 1B). In addition, some severely shrunken pollen also showed no obvious GFP fluorescence signal, and it is speculated that the transgenic pollen inclusion has been completely degraded, resulting in the inability to observe the fluorescence signal. These results suggested that the heterologous expression of *BcMF30a* or *BcMF30c* in Arabidopsis was indeed closely related to abnormal pollen development and can lead to different pollen phenotypes in the same anther.

### 2.2. Identification of Three Types of Transgenic Plants Expressing BcMF30a or BcMF30c

**Figure 2:**
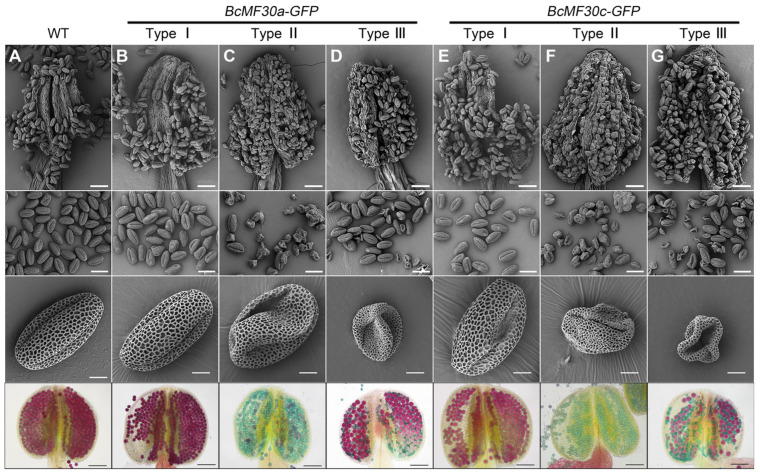
Scanning electron micrograph (SEM) observation and Alexander staining of mature pollen grains from different types of BcMF30a-GFP and BcMF30c-GFP transgenic plants of *Arabidopsis thaliana*. Pollen grains from wild-type (WT; (**A**)), Type I (**B**,**E**), Type II (**C**,**F**), and Type III (**D**,**G**) transgenic plants of *BcMF30a-GFP* (**B**–**D**) and *BcMF30c-GFP* (**E**,**F**). Bars = 50 μm, 30 μm, 5 μm, and 100 μm from the first row to the fourth row.

We next tried to isolate transgenic plants with similar phenotypes of pollen in the same anther from the T_1_ generation so as to systematically observe and analyze the impacts of heterologous expression of *BcMF30a* or *BcMF30c* in Arabidopsis on pollen development. Finally, we isolated three types of transgenic plants from a large number of T_2_ and T_3_ generations of *BcMF30a-GFP* and *BcMF30a-GFP* plants: Type I homozygous transgenic plants, producing highly vigorous mature pollen with normal morphology, which are not significantly different from wild-type plants (Figure 2A,B,E); Type II homozygous transgenic plants, generating collapsed pollen grains with no or low viability, but they are normal in size (Figure 2C,F); and Type III heterozygous transgenic plants, producing half normal pollen and half abortive pollen, which are obviously smaller than that of wild-type pollen (Figure 2D,G). The inability to obtain homozygous Type III plants implied that the transgenic pollens in Type III plants were completely aborted.

**Figure 3:**
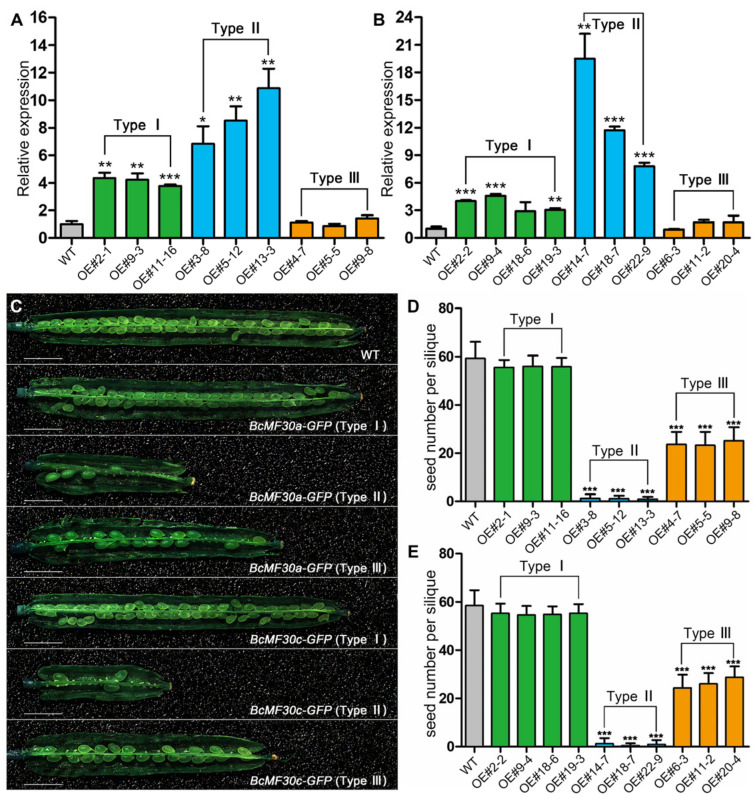
*BcMF30a-GFP* and *BcMF30c-GFP* transgenic plants with different gene expression levels show different numbers of seeds per silique. Analysis of gene expression levels in inflorescence of different types of *BcMF30a-GFP* (**A**) and *BcMF30c-GFP* (**B**) transgenic plants. *TUB4* was used as a reference gene, and the gene expression level in wild-type (WT) plants was set as 1. Values are mean ± SD. (**C**) Representative images of fully developed siliques from different types of *BcMF30a-GFP* and *BcMF30c-GFP* transgenic plants. Bars = 2 mm. The number of seeds per silique of different types of *BcMF30a-GFP* (**D**) and *BcMF30c-GFP* (**E**) transgenic plants. Error bars represent SD, n = 30. Asterisks on columns (**A**,**B**,**D**,**E**) indicate statistically significant differences from WT calculated using Student’s *t* test: *, *p* ≤ 0.05, ** *p* ≤ 0.01; *** *p* ≤ 0.001.

Since BcMF30a, BcMF30c, and their homologous protein (AtC3H18) in Arabidopsis exhibit more than 82% identity, we designed a pair of primers to detect the expression of the total mRNA of *BcMF30a* and *AtC3H18*, or *BcMF30c* and *AtC3H18*, in the inflorescence of transgenic plants. For both *BcMF30a-GFP* and *BcMF30c-GFP* plants, the qRT-PCR results showed that the expression levels of transcripts in Type I and Type II floral buds were significantly higher than that of the wild type, but there was no difference between Type III and the wild type (Figure 3A,B). Interestingly, there was also a general higher expression level in Type II inflorescence than in Type I (Figure 3A,B).

**Figure 4:**
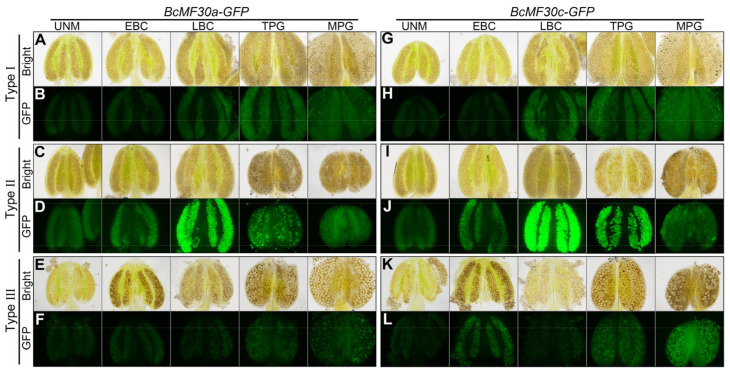
The development and GFP fluorescence signals of anthers from *BcMF30a-GFP* and *BcMF30c-GFP* transgenic plants. White field images (Bright) and epifluorescence images (GFP) of anthers from Type I (**A**,**B**,**G**,**H**), Type II (**C**,**D**,**I**,**J**), and Type III (**E**,**F**,**K**,**L**) transgenic plants of *BcMF30a-GFP* (**A**–**F**) and *BcMF30c-GFP* (**G**–**L**). UNM, uninucleate microspore; EBC, early-bicellular pollen; LBC, late-bicellular pollen; TCP, immature tricellular pollen; MPG, mature pollen grain. Bars = 100 μm.

All the transgenic plants were visually indistinguishable from the wild type, except for siliques (Figure 3C and Supplementary Figure S1). As expected, the number of seeds harbored in the siliques of Type I plants was not significantly reduced compared to the wild type. However, in Type II plants, there were, on average, less than five seeds per silique. The number of seeds in Type III siliques was also reduced to only about half of that of the wild type (Figure 4D,E). Nevertheless, the pollination test indicated that the heterologous-expressed *BcMF30a* and *BcMF30c* did not affect pistil development, since fertile pollen grains can germinate and grow normally in the pistils of all types of *BcMF30a-GFP* and *BcMF30c-GFP* transgenic plants (Supplementary Figure S2). These results suggested that the decrease in seed sets in Type II and Type III transgenic plants of both *BcMF30a-GFP* and *BcMF30c-GFP* was caused by the pollen abnormality.

### 2.3. Pollen Abortion Occurs at Different Pollen Developmental Stages in Different Types of Transgenic Plants

**Figure 5:**
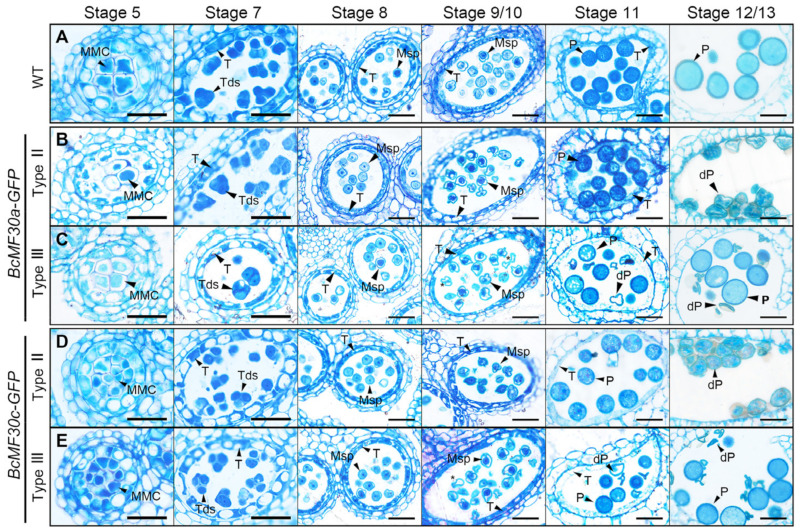
Semi-thin transverse sections of anthers from *BcMF30a-GFP* and *BcMF30c-GFP* transgenic plants. The semi-thin sections of anthers (stages 5, 7, 8, 9/10, 11, and 12/13) from wild type (WT; (**A**)), Type II (**B**,**D**), and Type III (**C**,**E**) transgenic plants of *BcMF30a-GFP* (**B**,**C**) and *BcMF30c-GFP* (**D**,**E**). Asterisks in (**C**,**E**) indicate microspores with less cytoplasm. MMC, microspore mother cell; Msp, microspore; P, pollen; dP, degenerated pollen; T, tapetum; Tds, tetrads. Bars = 25 μm.

**Figure 6:**
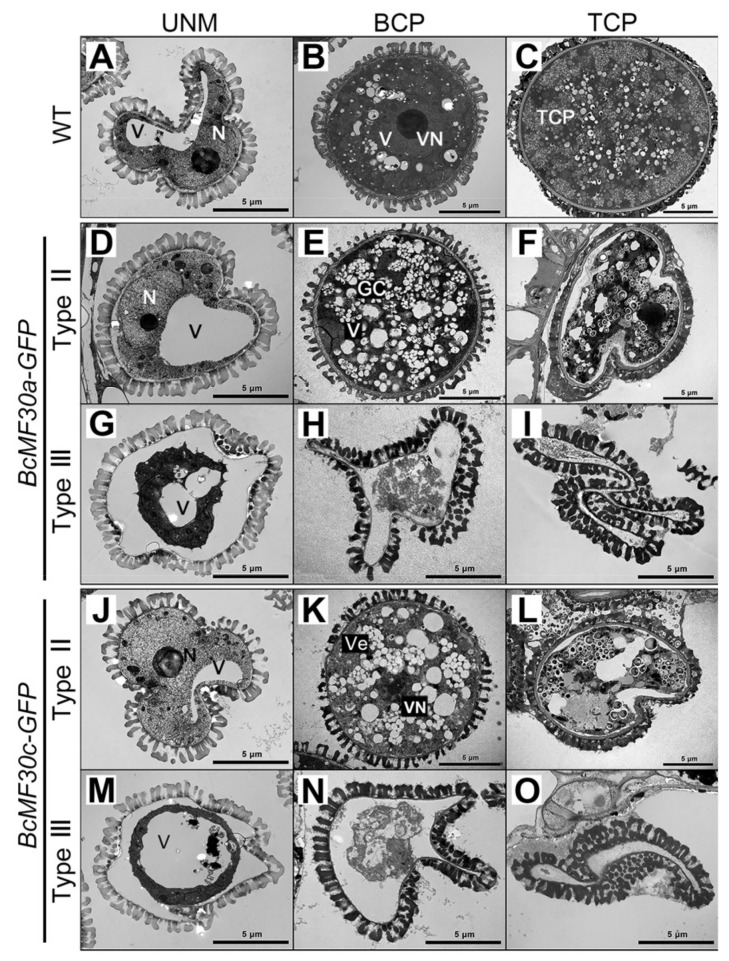
Transmission electron micrographs (TEMs) of pollen grains from *BcMF30a-GFP* and *BcMF30c-GFP* transgenic plants. Ultrastructure of pollen grains at different developmental stages from wild-type (WT; **A**–**C**), Type II (**D**–**F**,**J**–**L**), and Type III (**G**–**I**,**M**–**O**) transgenic plants of *BcMF30a-GFP* (**D**–**I**) and *BcMF30c-GFP* (**J**–**O**). BCP, bicellular pollen; GC, generative cell; N, nucleus; TCP, tricellular pollen; UNM, uninucleate microspores; V, vacuole; Ve, vesicle; VN, vegetative nucleus. Bars = 5 μm.

To investigate the pollen development process of different types of plants and determine the period when Type II and Type III transgenic pollen began to develop abnormally, we monitored the entire process of microspores’ development into mature pollen grains in different types of *BcMF30a-GFP* and *BcMF30c-GFP* plants. The following results showed that the pollen phenotypes of the heterologous expression of BcMF30a or BcMF30c in Arabidopsis were approximately the same (Figure 4, Figure 5 and Figure 6).

First, light microscopy and fluorescence microscopy were used to observe the plumpness of the anthers and the fluorescence intensity of pollen grains in three types of *BcMF30a-GFP* and *BcMF30c-GFP* plants, respectively (Figure 4). The bright images showed that the anthers of Type I plants always remained in a plump state throughout the pollen development (Figure 4A,G), similar to the wild type (Supplementary Figure S3A). Type II anthers showed obvious signs of shrinking at the trinucleate stage, and a serious collapse could be clearly observed at the mature pollen stage (Figure 4C,I). In type III plants, pollen with different plumpness can be simultaneously observed in the same anthers at the early-binucleate stage, and there were both plump mature pollen grains and deformed pollen at the mature pollen stage (Figure 4E,K). The fluorescence images of anthers revealed the following points and explained some questions: (1) Consistent with our previous reported results [41,42], the GFP fluorescence signals in anthers showed that BcMF30a and BcMF30c were expressed only in pollen and highly expressed in bicellular pollen, but not in other anther structures (Figure 4B,H). (2) According to the fluorescence intensity, the expression levels of fusion proteins in Type II transgenic pollen were significantly higher than that in Type I transgenic pollen, which was consistent with the results of qRT-PCR (Figure 3A,B, and Figure 4B,D,H,J). (3) Most of the plump pollen grains in Type III anthers showed no fluorescent signal, indicating that they were wild-type pollen, whereas the other half of the pollen grains showed signs of abortion at the early-binucleate stage, which explains the relatively low mRNA expression levels in Type III floral buds detected by qRT-PCR (Figure 3A,B and Figure 4F,L). As a control, only weak autofluorescence signals were detected in wild-type anthers (Supplementary Figure S3A).

A semi-thin transverse section analysis was performed to further clarify the anther development process. The results showed that no detectable difference between wild-type and Type II anthers were observed until stage 11, during which the bicellular pollen grains were produced. Although the pollen size seemed normal in Type II pollen at this stage, its cytoplasm showed to be more inhomogeneous than that of wild-type pollen. At stage 12/13, these pollen grains were severely deformed due to atrophy (Figure 5B,D). In Type III anthers, the more severely noticeable defects were first observed at stage 9/10. Some microspores seemed to contain less cytoplasm and were slightly stained, and about half of the pollen grains were degraded at stage 11 and completely shriveled at stage 12/13 (Figure 5C,E).

To further characterize the degradation phenotype of transgenic pollen, its ultrastructure was analyzed using TEM. The Type II microspore seemed to be normal at the late-uninucleate stage (Figure 6D,J). However, the shape of the Type III transgenic microspore was irregular and showed no obvious nucleus, and the cytoplasm was starting to degrade (Figure 6G,M). During the binucleate stage, unlike wild-type pollen, which contained two nuclei, only a generative nucleus was present in Type II bicellular pollen, and no discernible vegetative nucleus was observed. Meanwhile, many small vacuoles spread throughout the cytoplasm (Figure 6B,E,K). As expected, the contents of Type III pollen were further degraded and progressively disappeared at the binucleate stage, and the remaining internal structures were no longer distinguishable (Figure 6H,O). By the trinucleate stage, wild-type pollen grains contained well-developed dense cytoplasm, while Type II tricellular pollen was severely deformed due to the degeneration of cytoplasm, leaving only a small amount of inclusion (Figure 6C,F,L). Type III transgenic pollen, at this stage, completely collapsed and was devoid of cellular contents (Figure 6I,P).

In brief, three types of BcMF30a-GFP and BcMF30c-GFP transgenic plants showed different pollen development processes, and Type II and Type III transgenic pollen began to abort at different stages: the pollen development of Type I plants was always normal; Type II pollen showed obvious abnormalities at the binucleate stage; and in Type III transgenic plants, the abnormal development of transgenic pollen began from the late-uninucleate stage, earlier than that of Type II pollen.

### 2.4. Continuous Assembly of Cytoplasmic Foci Containing BcMF30a or BcMF30c Impairs Pollen Development

**Figure 7:**
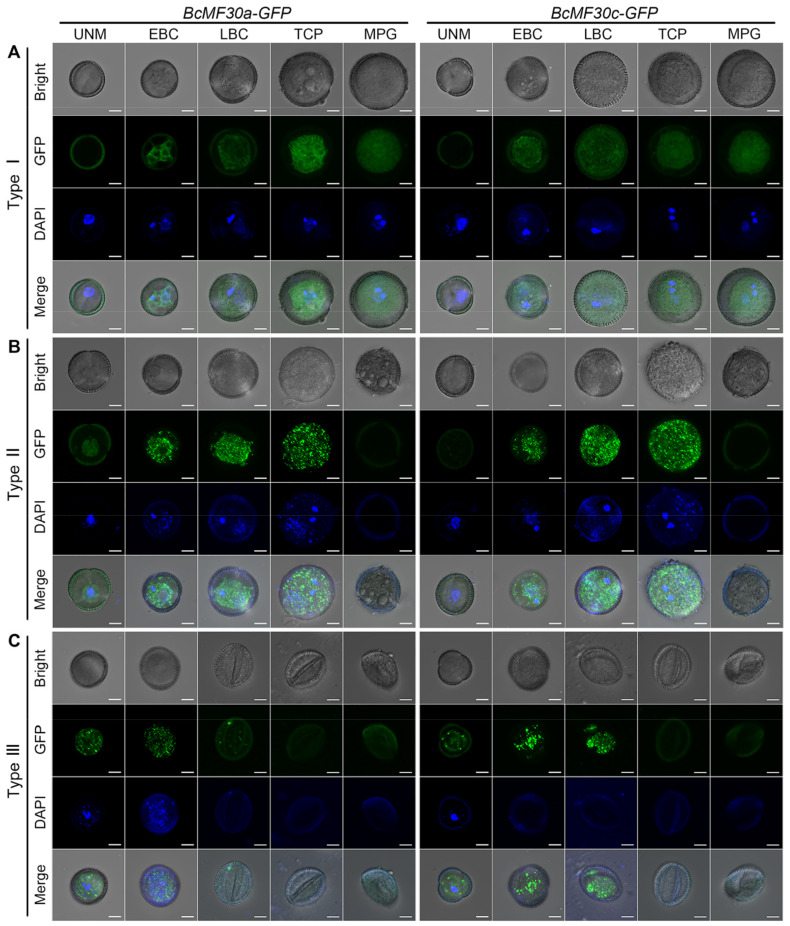
High expression of BcMF30a or BcMF30c in pollen grains induces the assembly of a large number of cytoplasmic foci, which disrupt pollen development. The GFP fluorescence signals and the results of DAPI staining test of Type I (**A**), Type II (**B**), and Type III (**C**) transgenic pollen grains from *BcMF30a-GFP* and *BcMF30c-GFP* plants. UNM, uninucleate microspore; EBC, early-bicellular pollen; LBC, late-bicellular pollen; TCP, immature tricellular pollen; and MPG, mature pollen grain. Bars = 5 μm.

The above results demonstrated that the heterologous expression of *BcMF30a* or *BcMF30c* can lead to different pollen phenotypes. Interestingly, when observing the transgenic pollen expressing BcMF30a-GFP or BcMF30c-GFP, it could be found that GFP fluorescence signals in some pollen grains seemed to present a punctate distribution, especially during the uninucleate and binucleate stages (Figure 1C), suggesting that BcMF30a and BcMF30c may exhibit special subcellular localization under certain circumstances. So, is there a causal relationship between the subcellular localization of these two proteins in pollen and different pollen phenotypes? To explore this question, the distribution of BcMF30a-GFP and BcMF30c-GFP fusion proteins in transgenic pollen at various developmental stages and the development of pollen nuclei were examined in detail (Figure 7)

For both *BcMF30a-GFP* and *BcMF30c-GFP* plants, Type I mature pollen grains were morphologically normal, with well-developed pollen nuclei, which were not different from that of wild-type pollen (Supplementary Figure S3B). Although the fusion proteins presented a non-uniform distribution in the bicellular and tricellular pollen grains, no large cytoplasmic foci were observed (Figure 7A). In Type II plants, pollen did not collapse until the mature pollen stage. However, the vegetative nucleus of Type II early-bicellular pollen appeared to be scattered, which was significantly different from the compact vegetative nucleus in wild-type pollen. Moreover, this scattered vegetative nucleus was subsequently degraded. Although the generative nucleus could successfully divide to form two sperm nuclei, these nuclei disappeared at the mature pollen stage. Remarkably, a lot of cytoplasmic foci containing BcMF30a-GFP or BcMF30c-GFP proteins were detected both in bicellular and tricellular pollen grains (Figure 7B). In Type III plants, distinguishable cytoplasmic foci were first observed in transgenic microspores, and the number of foci increased in early-bicellular pollen. Correspondingly, the nucleus in the microspore failed to develop and differentiate into a vegetative nucleus and a generative nucleus (Figure 7C). Together, these findings indicate that the cytoplasmic foci induced by the overexpression of BcMF30a or BcMF30c are detrimental to pollen development.

### 2.5. BcMF30a and BcMF30c Can Be Recruited into Cytoplasmic Foci like PBs and SGs

Several studies have shown that all AtTZFs and multiple other CCCH zinc-finger proteins can be localized in cytoplasmic foci similar to PB and SG [26,29,32,33]. Our previous study also showed that BcMF30a and BcMF30c can form cytoplasmic foci in tobacco epidermal cells after heat treatment [42]. In order to investigate whether BcMF30a and BcMF30c were associated with PB and/or SG, the co-localization of BcMF30a and BcMF30c with Arabidopsis PB and SG marker proteins, DCP2 and PABP8, was implemented by using a tobacco transient expression system.

**Figure 8:**
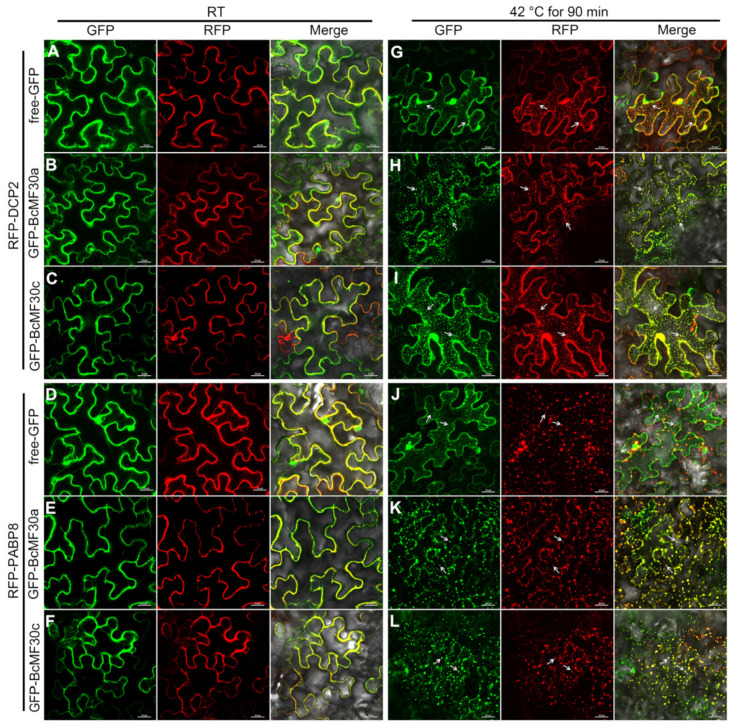
BcMF30a and BcMF30c can co-localize with PB and SG marker proteins in *Nicotiana benthamiana* leaf epidermal cells after heat treatment. When GFP was translationally fused to the N-terminus of BcMF30a (**B**,**E**,**H**,**K**) and BcMF30c (**C**,**F**,**I**,**L**), similar subcellular localization patterns were observed that BcMF30a and BcMF30c were diffused in cytoplasm at room temperature (RT) (**B**,**C**,**E**,**F**) and can co-localize with DCP2 and PABP8 after heat treatment (**H**,**I**,**K**,**L**). DCP2 and PABP8 are marker proteins of PB and SG, respectively. No co-localization of free-GFP with DCP2 and PABP8 was observed at RT (**A**,**D**) or after heat treatment (**G**,**J**). Bars = 25 μm.

When BcMF30a and BcMF30c were co-expressed with RFP-DCP2 (PB marker protein) or RFP-PABP8 (SG marker protein) fusion proteins in tobacco epidermal cells, respectively, all proteins were predominantly diffused in cytoplasm at room temperature (RT; Figure 8B,C,E,F). After heat treatment, a near-perfect co-localization of BcMF30a or BcMF30c with DCP2 or PABP8 in granular structures was observed, regardless of whether GFP was fused to the N- (Figure 8H,I,K,L) or C-terminus (Supplementary Figure S4) of BcMF30a or BcMF30c. As a negative control, free-GFP was dispersed in cytoplasm and nuclei either at RT or after heat stress in the co-localization experiments (Figure 8A,D,G,J). These results demonstrated that both BcMF30a and BcMF30c exhibited a specific subcellular localization pattern in tobacco epidermal cells: diffused in the cytoplasm and nuclei under normal conditions, while aggregated into cytoplasm foci and able to associate with PB and SG during heat stress, indicating that these two CCCH zinc-finger proteins might be one of the protein components of PB and SG.

## 3. Discussion

Many researchers have investigated the involvement of CCCH zinc-finger protein genes in many processes of plant growth, development, and stress response [31,43]. In our previous study, we proposed that the proper expression levels of *BcMF30a* and *BcMF30c* during microgametogenesis are essential for normal pollen development in Brassica campestris [41,42]. However, we have not yet estimated the range of this proper expression level. Here, we were fortunate to screen and observe three types of pollen phenotypes caused by the heterologous expression of *BcMF30a* or *BcMF30c* in Arabidopsis. Transgenic plants showed normal pollen development when *BcMF30a* or *BcMF30c* were overexpressed by 2-to-6-fold (Type I plants; Figure 3A,B). However, when the genes were overexpressed by 6-to-14-fold (Type II *BcMF30a-GFP* plants; Figure 3A) or 6-to-24-fold (Type II *BcMF30c-GFP* plants; Figure 3B), pollen development abnormalities appeared from the early-binucleate stage, and pollen grains were completely aborted at the mature pollen grain stage. The qRT-PCR results of Type III plants cannot be compared with those of Type I and Type II plants, as Type III plants were heterozygous, and the contents of transgenic pollen were degraded during the microgametogenesis stage. Nevertheless, by observing the fluorescence intensity in uninucleate microspores (Figure 7), it can be inferred that the promoter activity of *BcMF30a* or *BcMF30c* promoters in Type III plants may be stronger than that in Type I and Type II plants. Correspondingly, the abnormal development of transgenic pollen in Type III plants started earlier and was also more severe than Type II pollen. Interestingly, transgenic plants overexpressing the homologous gene *AtC3H18* in Arabidopsis also exhibit similar phenotypes [32]. Hence, we conclude that both BcMF30a and BcMF30c and their homologous gene protein AtC3H18 may function in a dosage-dependent manner to participate in pollen development.

For most of the CCCH zinc-finger protein genes, especially *TZFs*, their biological functions were identified mainly based on the analysis of overexpression transgenic plants [31]. Interestingly, it was found that some transgenic plants showed some growth and development defects. For example, the overexpression of *AtTZF1*, *AtTZF4*, *AtTZF5,* or *AtTZF6* can cause leaves to become crinkled, making plants appear compact [30,44]. Some *AtTZF1* homozygous overexpression plants cannot even survive [44]. *AtTZF2* and *AtTZF3* overexpression lines displayed minor growth retardation at the early seedling growth stage [45]. However, there is currently no research to explain what causes these growth defects.

In this study, we also observed the adverse effects of the heterologous expression of *BcMF30a* or *BcMF30c* in Arabidopsis. Encouragingly, our study showed that the pollen abortion in transgenic plants was closely related to the localization of these two proteins in pollen during microgametogenesis (Figure 7). By observing the pollen phenotypes and monitoring the presence of fusion proteins in different types of transgenic pollen, we found the fact that the assembly of cytoplasmic foci containing BcMF30a-GFP or BcMF30c-GFP fusion proteins in pollen grains was always accompanied by pollen abortion. Moreover, the time when these foci began to form large numbers of cytoplasmic foci was highly consistent with the time when pollen development was blocked (Type II and Type III transgenic pollen). In contrast, there were no or only a few small cytoplasmic foci detected in the normally developed Type I transgenic pollen. These findings indicated that the continuous assembly of cytoplasmic foci due to the high expression of BcMF30a or BcMF30c was indeed the inducement of pollen abortion. In addition, these results also suggested that the formation of cytoplasmic foci was dependent on the high expression of BcMF30a or BcMF30c, implying that there may be a critical expression level to determine whether these foci can be successfully induced. In view of the fact that the transient expression of many CCCH proteins in protoplasts can form cytoplasmic foci [26], and some of them, such as AtTZF1 [29], AtC3H18 [32], and AtC3H18L [33], have even been shown to form foci when overexpressed in intact plants, we believe that the growth defects of the above-mentioned *TZF*-overexpressed plants may also be caused by the persistent formation of cytoplasmic foci due to the high expression of TZFs.

It is worth mentioning that, in addition to overexpression, the assembly of TZF-associated cytoplasmic foci can also be induced by hormone treatment and some stresses. In Arabidopsis, jasmonic acid (JA) treatment can induce the formation of AtTZF1-associated cytoplasmic foci [29]. Similarly, JA-induced foci could be observed in *AtTFZ6*-overexpressing plants, driven by its native promoter [30]. ABA and salt stress could promote the assembly of OsTZF1-associated cytoplasmic foci, which was predominantly localized in the cytoplasm under native protein levels [46]. Interestingly, many TZFs are involved in hormone response (e.g., ABA, gibberellin, and JA), and their overexpression usually enhances the resistance of transgenic plants to certain stresses [31]. For instance, *AtTZF1*-overexpression transgenic plants were superior to wild-type plants in regard to drought and cold tolerance [44]. The overexpression transgenic plants of *AtTZF2*, *AtTZF3*, *AtTZF10*, *AtTZF11*, *OsTZF1,* and *GhZFP1* all showed enhanced tolerance to salt stress [45,46,47,48,49,50]. Therefore, it is tempting to assume that there is a causal relationship between the assembly of TZF-associated cytoplasmic foci induced by hormone treatment or stresses and the enhancement of resistance.

Unlike overexpression, which usually induces the persistent assembly of cytoplasmic foci, hormone treatment or stress only lasts for a short period of time, which means that these foci cannot be continuously formed. Intriguingly, studies have shown that the assembly of some mRNP granules is reversible, and their components (e.g., mRNAs) can be released into cytoplasm and reused [19,24]. In yeast, PBs were formed to transiently preserve a pool of non-translating mRNAs during glucose starvation, allowing these mRNAs to restore translation activity upon glucose re-addition, which endows yeast with better ability to cope with short-term glucose starvation [51]. In Arabidopsis, mRNAs encoding ribosomal proteins can be preferentially stored in SGs during heat shock, and they can be released and translated during recovery [19]. Previously, we also revealed that the assembly of mRNP granules containing AtC3H18 is highly dynamic and reversible, and it depended on the availability of mRNA [32]. Here, we showed that both BcMF30a and BcMF30c can co-localized with marker proteins of PBs and SGs during heat stress, indicating that (1) these two proteins are potential components of PBs and SGs; (2) these foci may have functions of temporarily storing translationally inhibited mRNAs, thereby participating in mRNA metabolism, like PBs and SGs; and (3) BcMF30a/BcMF30c-associated cytoplasmic foci may be involved in heat stress response. If these cytoplasmic foci are confirmed to be mRNP granules that can store mRNAs, it can be speculated that the abortion of Type II and Type III transgenic pollen may be caused by the continuous formation of cytoplasmic foci that results in a large number of mRNAs to be trapped and cannot be re-translated in time. In contrast, TZFs-associated cytoplasmic foci induced by a short-term hormone or stress treatment may still retain the ability to disassemble, allowing the mRNAs stored in them to be reused. This may be one of the hypotheses explaining why the moderate overexpression (possibly below the critical value of inducing cytoplasmic foci) of TZFs can strengthen the resistance to specific stress. Therefore, it will be the focus of our next work to determine whether BcMF30a/BcMF30c-associated cytoplasmic foci participate in gene expression regulation during microgametogenesis by storing mRNAs, and we plan to explore whether the moderate overexpression of *BcMF30a* or *BcMF30c* in pollen (i.e., Type I pollen) is conducive to enhancing the stress resistance of pollen.

## 4. Materials and Methods

### 4.1. Plant Material and Growth Conditions

*Arabidopsis thaliana* (Columbia-0) was used for heterologous genetic transformation. T_1_-lines transgenic Arabidopsis seedlings were screened on synthetic Murashige and Skoog (MS) medium containing 50 mg·L**^−^**^1^ kanamycin in a 22 °C growth chamber under a 14 h light/10 h dark regime. All Arabidopsis plants were planted in a phytotron at 20 ± 2 °C, under a 16/8 h light/dark cycle. Tobacco (*Nicotiana benthamiana*) plants were grown in the growth room at 26 ± 2 °C, under a 16/8 h light/dark cycle.

### 4.2. Generation of BcMF30a-GFP and BcMF30c-GFP Transgenic Arabidopsis Plants

To construct the vectors that express BcMF30a-GFP and BcMF30c-GFP fusion proteins, *GUS* reporter genes in the recombinant vectors *ProBcMF30a:BcMF30a* and *ProBcMF30c:BcMF30c* that were constructed in our previous study [42] were replaced by *GFP*. Then, the recombinant plasmids *ProBcMF30a:BcMF30a-GFP* and *ProBcMF30c:BcMF30c-GFP* were introduced into Arabidopsis by a standard floral dipping method. The transgenic plants were screened on MS/agar medium and further identified by PCR and sequencing. The primers used are listed in Supplementary Table S1.

### 4.3. RNA Extraction and qRT-PCR

Total RNA extraction, cDNA preparation, and qRT-PCR were conducted as described by Xu et al. [41]. *TUB4* was selected as reference genes to normalize the quantity of total RNA. All primers used are listed in Supplementary Table S1.

### 4.4. Phenotypic Analyses, Cytological Observation, and Pollen Germination Assay

Alexander solution and 4′,6-diamidino-2-phenylindole (DAPI) solution were used to investigate the pollen vigor and the development of nuclei in pollen, respectively. Scanning electron microscopy (SEM), semi-thin transverse section analysis, transmission electron microscopy (TEM) analysis, and in vivo pollen germination tests were performed as described by Lin et al. [52] and Xu et al., but with some modifications. The images of Alexander-stained anthers, fertilized pistils, and anthers at different developmental stages were photographed by using a fluorescent microscope (ECLIPSE 90i, Nikon Corp., Tokyo, Japan). The micrographs of DAPI-stained pollen grains were captured in a confocal laser scanning microscope (A1, Nikon, Japan), using the ND Acquisition tool (NIS-Elements AR software) with Z Movement.

### 4.5. Subcellular Localization

Plasmids for subcellular co-localization experiments were constructed in our previous studies, namely *pFGC-BcMF30a/c-GFP*, *pFGC-GFP-BcMF30a/c* [41], *pFGC-RFP-DCP2,* and *pFGC-RFP-PABP8* [33]. To investigate the co-localization of BcMF30a/c and DCP2 or PABP8, Agrobacterium-containing vectors expressing GFP-fusion proteins and RFP-fusion proteins were mixed in a ratio of 1:1 and transiently transformed into tobacco leaf epidermal cells. About 48 h later, the co-localization of fusion proteins in tobacco leaf epidermal cells at RT and under heat treatment (42 °C for 90 min) was analyzed under a confocal laser scanning microscope (A1, Nikon, Japan), using ND Acquisition tool (NIS-elements AR software) with or without Z Movement.

## Data Availability

All the necessary data generated are provided in the form of figures, tables, and Supplementary Materials.

## Supplementary Materials

The following supporting information can be downloaded at https://www.mdpi.com/article/10.3390/ijms242316862/s1.
