# Supplementary material for: Heterologous Expression of Two Brassica campestris CCCH Zinc-Finger Proteins in Arabidopsis Induces Cytoplasmic Foci and Causes Pollen Abortion
Source: Int J Mol Sci. 2023 Nov 28;24(23):16862. doi: 10.3390/ijms242316862 (PMC10706035; doi:10.3390/ijms242316862)
Supplement: Supplementary file 1 [file ijms-24-16862-s001.zip › ijms-2651338-supplementary.pdf]

Supplementary Materials

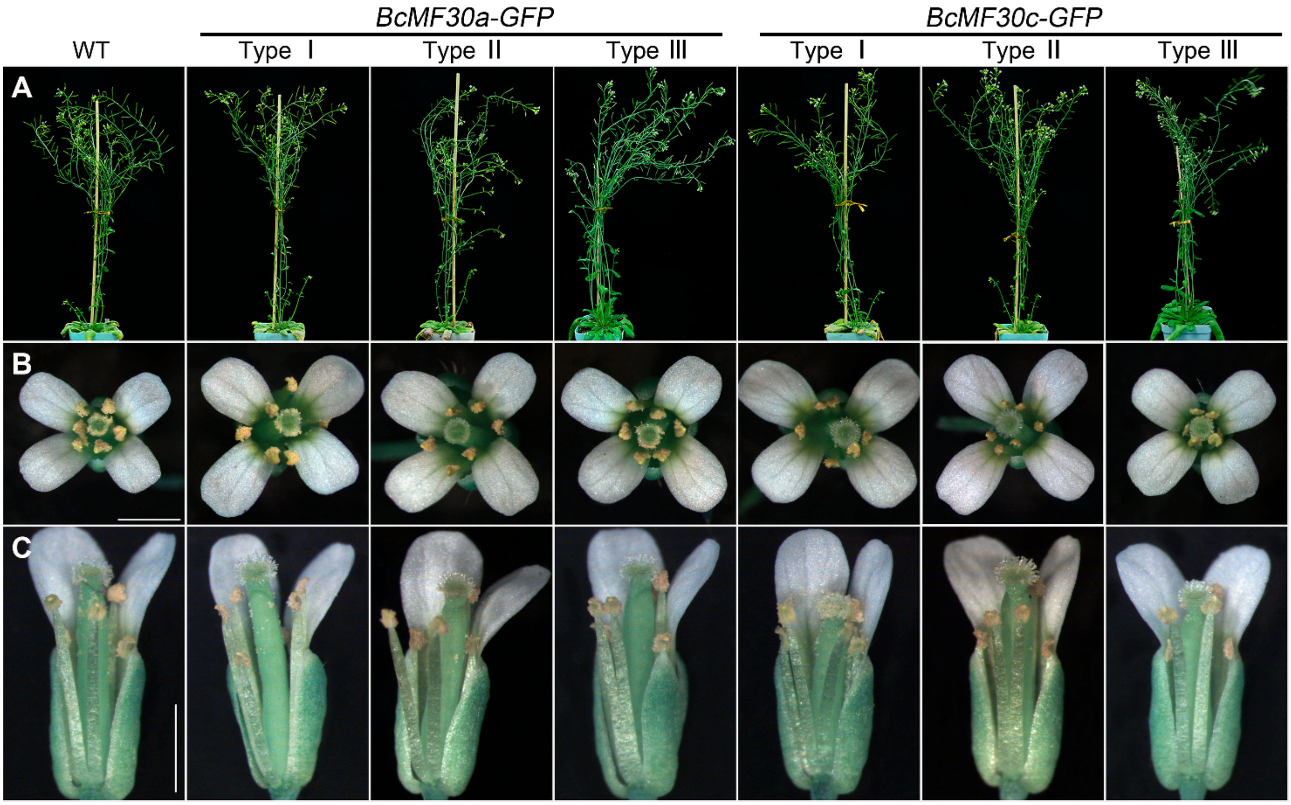

Figure S1. Heterologous expression of *BcMF30a* and *BcMF30c* in Arabidopsis does not affect the morphology of plants and floral organs. (**A**) Morphological observation of different types of *BcMF30a-GFP* and *BcMF30c-GFP* transgenic plants. Top views (**B**) and side views (**C**) of flowers from different types of *BcMF30a-GFP* and *BcMF30c-GFP* transgenic plants. Bars = 1mm in (**B, C**).

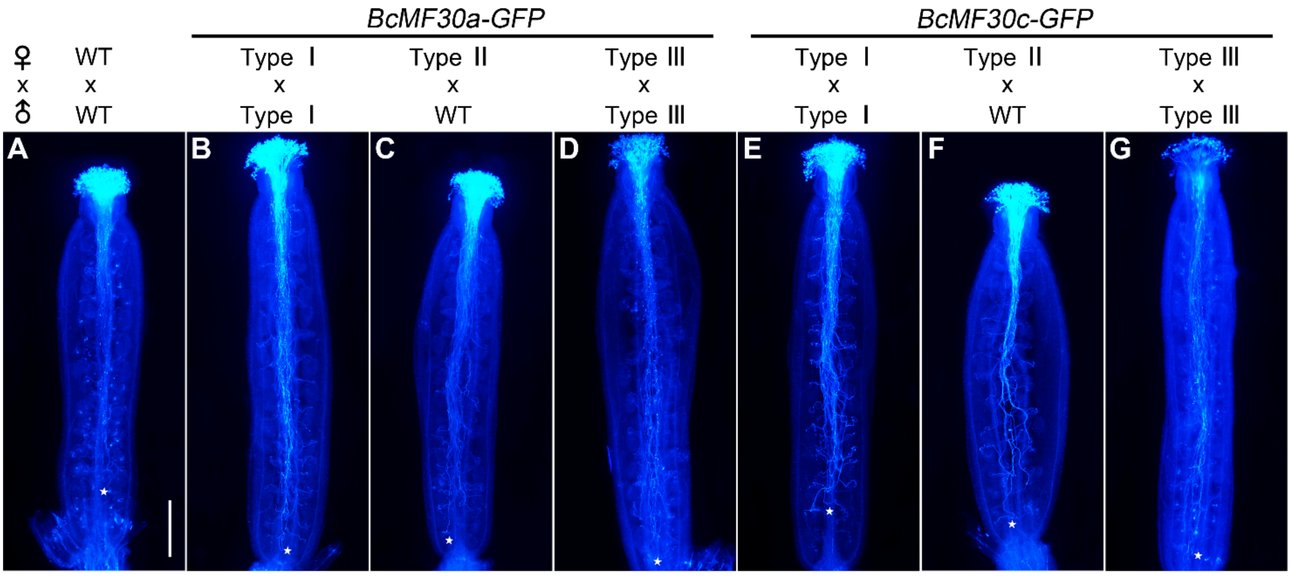

Figure S2. *In vivo* pollen germination tests showed that the pistils of all types of *BcMF30a-GFP* and *BcMF30c-GFP* transgenic plants developed normally. Self-pollination or wild-type pollen grains pollinated on the pistils of transgenic plants for 24 h. The asterisks indicated the end position of pollen tubes. Bars = 100 μm.

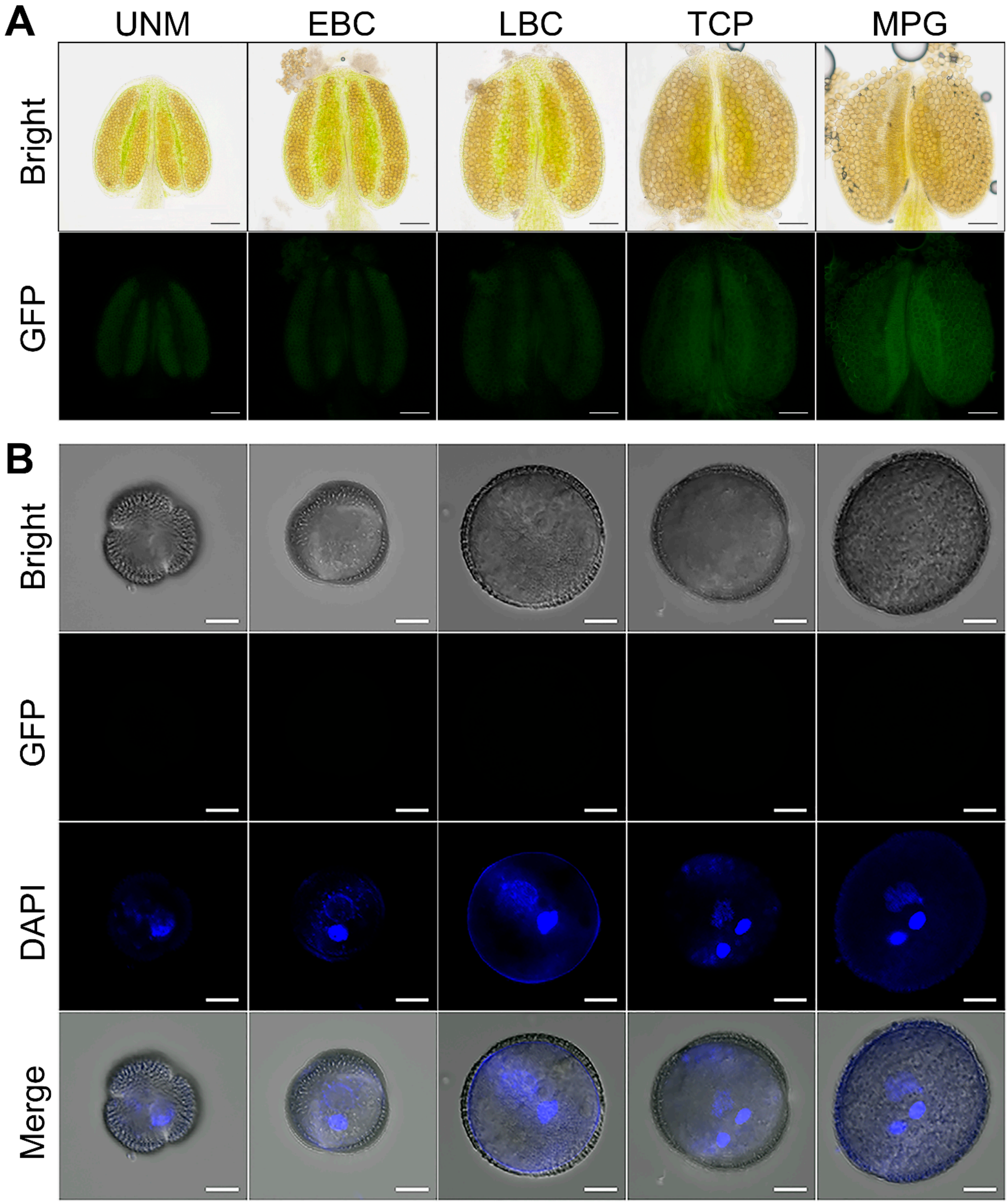

Figure S3. The anther development (**A**) and pollen development (**B**) of wild-type plants. UNM, uninucleate microspore; EBC, early bicellular pollen; LBC, late bicellular pollen; TCP, immature tricellular pollen; and MPG, mature pollen grain. Bars = 100 μm in (**A**), 5 μm in (**B**).

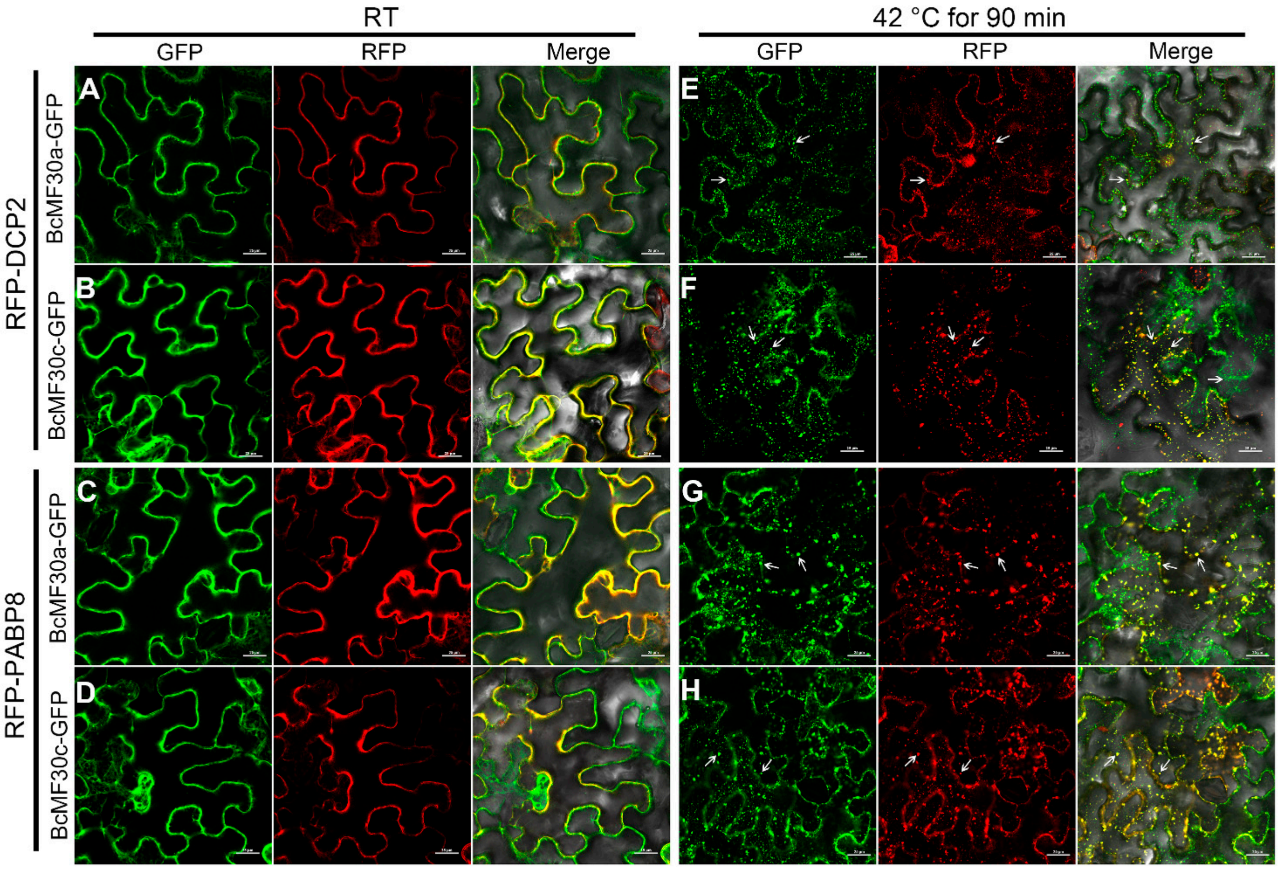

Figure S4. BcMF30a-GFP and BcMF30c-GFP can co-localized with PB and SG marker proteins in *Nicotiana benthamiana* leaf epidermal cells after heat treatment. DCP2 and PABP8 are marker proteins of PB and SG, respectively. Both BcMF30a-GFP and BcMF30c-GFP were dispersed in cytoplasm at room temperature (RT) when co-expressed with RFP-DCP2 (**A,B**) and RFP-PABP8 (**C,D**), respectively. After heat treatment at 42°C for 90 min, both BcMF30a-GFP and BcMF30c-GFP formed cytoplasmic foci and co-localized with RFP-DCP2 and RFP-PABP8 (**E–H**). Pictures represent white field images (Bright), epifluorescence (GFP and RFP) and merged images (Merge). Bar =25 μm.

Table S1 Sequences of primers used in this study.

Experiment	Primer Name	Sequence-F(5'-3')	Sequence-R(5'-3')
GFP CDS	GFP-F/R	GACAGTTACCATGGTGAGCAAGGGCG	TGATCCCGGGGATCCCTAGGATCCCTTGTACAGCTC
NOS PCR	NOS-1F/R	TCTAGAGGATCCCCGGGATCAACAACTCTCCTGGCG	GACGGCCAGTGAATTCCCGATCTAG
pBI121(-GUS) Test	NOS-2F/R	AACGCAATTAATGTGAGTTAGC	GACGGCCAGTGAATTCCCGATCTAG
BcMF30a-GFP Test	BcMF30a-F/R	AGGACGGTTGTGCTATCT	TGTAAAACGACGGCCAGT
BcMF30c-GFP Test	BcMF30c-F/R	CGGAGAATCCATTGTGAA	TGTAAAACGACGGCCAGT
qRT-PCR	qRT-PCR-F/R	ATTACTCCTACCTCGGCTTCT	AACCGTTCTGCTTGTTCCT
TUB4 qRT-PCR	TUB4-F/R	AAGGCTTTCCTTCATTGGTACA	CTCTCCGGCTGTAGCATCTT
